# Predicting the evolution of pH and total soluble solids during coffee fermentation using near-infrared spectroscopy coupled with chemometrics

**DOI:** 10.1016/j.crfs.2024.100788

**Published:** 2024-06-17

**Authors:** Vicente Tirado-Kulieva, Carlos Quijano-Jara, Himer Avila-George, Wilson Castro

**Affiliations:** a Instituto de Investigación para el Desarrollo Sostenible y Cambio Climático, Universidad Nacional de Frontera, Sullana, 20100, Piura, Peru; b Escuela de Posgrado, Universidad Nacional de Trujillo, Trujillo, Peru; c Departamento de Ciencias Biológicas, Facultad de Ciencias Biológicas, Universidad Nacional de Trujillo, Trujillo, Peru; d Departamento de Ciencias Computacionales e Ingenierías, Universidad de Guadalajara, Ameca, 46600, Jalisco, Mexico; e Facultad de Ingeniería de Industrias Alimentarias y Biotecnología, Universidad Nacional de Frontera, Sullana, 20100, Piura, Peru

**Keywords:** Coffee, Feature selection, Fermentation, pH, NIRS

## Abstract

Currently, coffee fermentation is visually operated, which results in incomplete or excessive processes and coffees with undesirable characteristics. In front of it, pH and total soluble solids (TSS) have been shown to be good fermentation indicators, although this requires rapid, accurate, and chemical-free measurement techniques such as NIR spectroscopy. However, the complexity of the NIR spectra requires optimization steps in which variable selection techniques simplify profiles and subsequent models. This work tests a new covering array feature selection (CAFS) approach on NIR spectra to optimize prediction models in coffee samples during fermentation. Spectral profiles in the range 1100–2100 nm were extracted from coffee beans (Typica, Caturra, and Catimor varieties) raw and during fermentation (4, 8, 12, 16, 20, and 24 h). Partial least-squares regressions (PLSR) were performed using full spectra using a five-fold cross-validation strategy for training and validation. The relevant wavelengths were then selected using the *β* coefficients, the important projection of variables (VIP), and the CAFS method. Finally, optimized models were performed using the relevant wavelengths and compared among these using their statistical metrics. The models performed using the selected variables (22–47) of CAFS showed the best performance in predicting pH (*R*^2^ = 0.825–0.903, RMSE = 0.096–0.158, RPD = 6.33–10.38) and TSS (*R*^2^ = 0.865–0.922, RMSE = 0.688–1.059, RPD = 0.94–1.45) compared to the other methods. These findings suggest that simple and efficient models could be performed and implemented in routine analysis due to the maximum coverage and minimum cardinality of CAFS.

## Highlights

• Non-invasive coffee fermentation monitoring using NIR spectroscopy coupled with advanced chemometric techniques.
• TSS and pH, reliable indicators of fermentation progress, were modeled from NIR spectral data.
• Robust and accurate prediction models were developed using PLSR.
• The PLSR model optimized by CAFS showed superior performance in predicting TSS and pH.

## Graphical abstract

**Figure:**
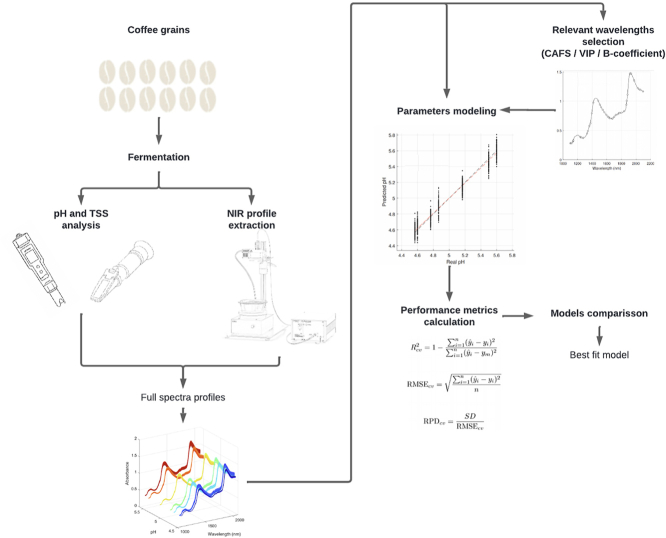

## 1 Introduction

Coffee (*Coffea* sp.) is the second most consumed commodity on the market after crude oil and the second most consumed beverage after water (Bosso et al., 2023). In 2021, around 10.9 million tons of coffee were produced with constant growth in demand and supply. Peru is the seventh largest producer of conventional coffee (FAO, 2024), in addition to being the first producer and exporter of organic coffee (MIDAGRI, 2022). Coffee is one of the main crops in the country, an important source of jobs and a key piece of the economy.

Fermentation is one of the stages that has the greatest influence on coffee quality, but its control is a challenge because the end of the process is determined visually or manually (Pereira and Moreira, 2021). This causes incomplete or excessive fermentations that induce unpleasant aromas and flavors (Pereira and Moreira, 2021). This was shown in several studies, such as in Zhang et al. (2019), where the quality of the cup decreased considerably when the coffee fermentation time was not optimal.

The capacity to monitor the fermentation of different parameters has been studied. Jackels and Jackels (2005) tested pH, glucose, lactic acid and ethanol, and it was established that pH was the only reliable indicator, a fact accepted by the scientific community (Córdoba-Castro and Guerrero-Fajardo, 2016). Similarly, Prakash et al. (2022) determined that total soluble solids (TSS) can indicate mucilage removal and fermentation progress. Therefore, pH and TSS can be used to determine the end of the process.

The determination methods for pH and TSS require trained personnel, chemicals for calibration, and significant time, especially when many samples must be tested, making it a tedious process (Velmourougane, 2013). Therefore, sensitive, rapid, nondestructive, multiparametric, and chemical-free techniques, such as near-infrared spectroscopy (NIRS), are required. NIRS measures the energy absorption by the sample exposed to electromagnetic radiation in the wavelength range of 750–2500 nm, collecting information on the spectral characteristics of hydrogen-containing functional groups (O–H, C–H, N–H and S–H) (Araújo et al., 2020; Castro et al., 2022). NIRS has been widely used in food analysis and has been effective in identifying the chemical properties of coffee, but has focused on the roasting process or ground coffee (de Carvalho Pires et al., 2021; Tugnolo et al., 2021).

A common characteristic of data obtained using spectroscopic techniques is the presence of numerous variables but few samples, which complicates the analytical problem. From a practical point of view, variable selection (VS) is important because it reduces noise and avoids overfitting, resulting in simpler models to facilitate data collection and interpretation without compromising their predictive capacity (de Araújo Gomes et al., 2022). This strategy also optimizes computational calculations and reduces hardware costs, which is desirable to guide the industry in the development of low-cost, compact, fast and lightweight tools (Fatemi et al., 2022). However, finding the most significant variables is challenging because no single method can extract the most essential and significant variables for a particular application (Kamruzzaman et al., 2022).

In food engineering applications, the effects of VS techniques such as the genetic algorithm (GA), the successive projection algorithm (SPA), the variable importance projection (VIP), stepwise regression (SWR), competitive adaptive reweighted sampling (CARS), and regression coefficients or *β* coefficients have been tested (Fatemi et al., 2022; Kamruzzaman et al., 2022). Since it is difficult to determine which VS algorithm is suitable for a specific type of data, it is necessary to compare and test different algorithms for various applications to select the best (Vásquez et al., 2018). The dynamic chemical complexity of food products during fermentation makes this challenge more difficult.

A good search strategy is needed for VS methods because it is hard to find a subset of wavebands that are good for predicting parameters during coffee fermentation (Castro et al., 2022). In this sense, covering arrays (CAs) come into play. CAs are mathematical objects that offer the advantages of maximum coverage and minimum cardinality, making them ideal for selecting a reduced set of variables that capture all relevant information in the data (Avila-George et al., 2012; Torres-Jimenez et al., 2019a). CAs have been used successfully in VS processes (Dorado et al., 2019; Solarte-Martinez et al., 2019; Villegas et al., 2018; Vivas et al., 2019). Our team just came up with a new CA-based VS method, called Covering Array Feature Selection (CAFS), that uses NIRS to tell the difference between Amazonian cacao-clone nibs (Castro et al., 2022).

This research looked at how CAFS, NIR spectra, and PLSR could be used together to guess the pH and TSS of coffee samples during fermentation and compared them to the *β* coefficients and VIP.

## 2 Material and methods

**Fig. 1:**
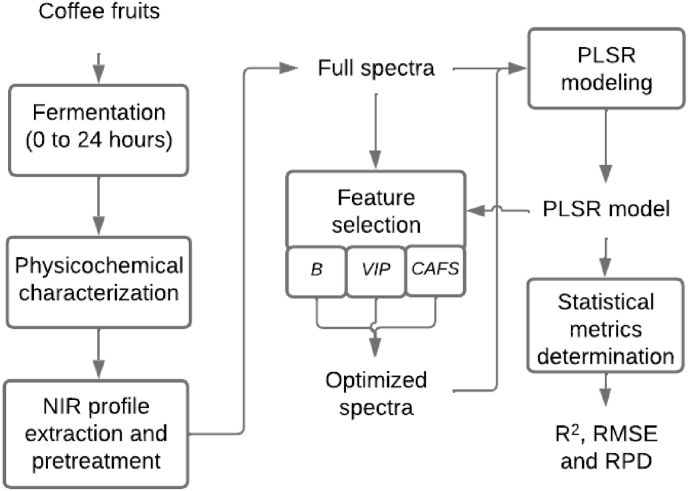
Proposed methodology for the study.

Fig. 1 shows the main steps of the methodology proposed in this study. Each step is detailed and commented on in the following subsections.

### 2.1 Coffee fermentation

Arabica coffee beans (*Coffea arabica* L.) var. Typica, Caturra, and Catimor were picked in June 2023 from the districts of Sícchez and Ayabaca in Piura, Peru. The fruits were transferred to the laboratory within 6 h after harvest, following the methodology of Elhalis et al. (2020). The green and dry fruits were removed and the selected fruits were pressed under aseptic conditions. Fermentation was carried out for 24 h with a dry must, which included coffee beans and a proportion of skin without additional water (Pereira et al., 2020).

The samples were collected at 0, 4, 8, 12, 16, 20, and 24 h from various locations within the prehomogenized mixture. Each sample was analyzed in duplicate and comprised approximately 100 defect-free grains of similar size. The grinding was carried out with a high-speed DAMAI HC-1000Y mill (Wuyi Haina Electric Appliance Co., Ltd., China) and the particle size was standardized to 300 μm using a sieve (Riceli Equipos, Peru).

### 2.2 Physicochemical characterization

The pH was measured directly in the volume of coffee with a pH meter (HANNA Instruments, Italy). A digital refractometer (HANNA Instruments, Italy) was used to measure TSS (°Brix) by taking a sample of liquid mucilage. All analyzes were performed in triplicate at room temperature and expressed as mean ± standard deviation.

Finally, the normality of the pH and TSS values was tested using the Kolmogorov-Smirnov test.

(1) $$D=maxD^{+},D^{-}$$

where $D^{+}=\max_{i}\left\{\frac{i}{n}-Z_{i}\right\}$, $D^{-}=\max_{i}\left\{Z_{i}-\frac{i-1}{n}\right\}$, *Z* = *F*(*X*_*i*_), *F*(*X*_*i*_) is the cumulative distribution function of the normal distribution, *X*_*i*_ is the *i*-th order statistic of a random sample, 1 < *i* < *n*, and *n* is the sample size.

### 2.3 NIR profile extraction and pretreatment

NIR spectral profiles were measured at room temperature. A Polytec PSS-A-T01 NIR spectrometer (Polytec GmbH, Germany), equipped with a tungsten halogen lamp as a light source and an InGaAs (Indium–Gallium–Arsenic) detector in the range of 1100–2100 nm, and a resolution of 2 nm, was used.

The database comprises 2100 profiles extracted from three coffee varieties, encompassing seven fermentation times and two sub-samples, with 50 profiles per sample. The NIR spectrometer was programmed to extract one profile at a time, with the sample remaining between measurements until fifty profiles were obtained.

Finally, the spectra acquired in reflectance mode were converted to absorbance according to Equation (2).

(2) $$A_{\lambda }=\log\left(\frac{1}{R_{\lambda }}\right)$$

where *A* is the absorbance, *λ* is the wavelength, and *R* is the reflectance.

Unwanted things such as noise, interference signals, sample heterogeneity, and baseline drift can change spectral profiles, so spectral corrections are needed (Vásquez et al., 2018). The NIR spectra were smoothed using the Savitzky-Golay filter, whose parameters were second-order and three-step windows; see Equation (3).

(3) $$y_{j}^{o}=\frac{\sum_{i=-m}^{m}C_{i}Y_{j+i}}{N}$$

where *Y* is the original profile, *y*^*o*^ the smoothed profile, *C* is the coefficient of the *i*-th term of the profile, and *N* is the number of convolution stages.

### 2.4 PLSR modeling

All wavelengths were used to build partial least squares regression (PLSR) models to predict the pH and TSS of the coffee samples of each variety. PLSR was chosen as the test model because, in conjunction with spectroscopic techniques, it is one of the most widely used multivariate regression techniques in food analysis (Vásquez et al., 2018).

PLSR transforms predictor variables (*X*) into response variables (*Y*). PLSR decomposes *X* and *Y* to project them in new directions and describe the change of the variables together to the greatest extent possible (Vásquez et al., 2018). Then a regression step is performed with the decomposed *X* and *Y*, whose model is shown in Equation (4):

(4) Y=βX+e

where *Y* represents the pH or TSS values of the coffee, *X* is the absorbance data matrix (*n* observations × *m* wavelengths), and *β* is the coefficient matrix.

### 2.5 Feature selection

Removal of irrelevant spectral information allows us to obtain simplified and practical models, which require revealing the most important spectral bands for modeling. The methods tested are commented on below:

● *β* coefficients or Regression coefficients (BC). They are unique measures of association between each variable and the response. The wavelengths are related to the absolute load weights of the entire PLSR model and are selected according to their value and predictive ability (Vásquez et al., 2018).
● Variable Importance in Projection (VIP). The VIP, represented as *v*_*j*_, is a measure of the contribution of each variable according to the variance explained by each component of PLSR, as shown in Equation (5) (Mehmood et al., 2020).

(5) $$v_{i}=\sqrt{p\overset{A}{\underset{a=1}{\sum }}\left[\frac{SS_{a}{\left(w_{aj}/\|w_{a}\|\right)}^{2}}{\sum_{a=1}^{A}SS_{a}}\right]}$$

where *p* is the number of variables, *SS*_*a*_ is the sum of squares explained by the *a*-th component, and ${\left(w_{aj}/\|w_{a}\|\right)}^{2}$ represents the importance or weight of the *j*-th variable. Variables with VIP values greater than 1 were considered informative variables.

● CAFS. Covering arrays (CAs) are defined as a matrix of *N* rows and *k* columns over an alphabet of *v* symbols, such that for each set of *t* columns, each tuple of symbols is covered at least once, denoted by CA(*N*; *t*, *k*, *v*) (Torres-Jimenez et al., 2018, 2019b). More details about this variable selection method are provided in Castro et al. (2022).

### 2.6 Statistical metrics determination

The models were trained using a *K*-Fold cross-validation strategy (*K* = 5) carried out with 30 repetitions. In each repetition, the following performance statistical metrics were determined: coefficient of determination (*R*^2^), root mean square error (RMSE), and the ratio of performance to deviation (RPD) defined by Equations (6), (7), (8)).

(6) $$R_{cv}^{2}=1-\frac{\sum_{i=1}^{n}{\left({\hat{y}}_{i}-y_{i}\right)}^{2}}{\sum_{i=1}^{n}{\left({\hat{y}}_{i}-y_{m}\right)}^{2}}$$

(7) $${\text{RMSE}}_{cv}=\sqrt{\frac{\overset{n}{\underset{i=1}{\sum }}{\left({\hat{y}}_{i}-y_{i}\right)}^{2}}{n}}$$

(8) $${\text{RPD}}_{cv}=\frac{SD}{{\text{RMSE}}_{cv}}$$

where ${\hat{y}}_{i}$ and *y*_*i*_ are the predicted and reference response variables of the *i*-th sample, *y*_*m*_ is the mean value of the samples, *n* is the number of samples, and *SD* is the standard deviation of *y*. The subscript *cv* refers to the calculations performed in the cross-validation strategy.

The processing of spectral data, the development of the models, and the selection of relevant variables, functions, and routines were implemented in Matlab R2023a (The MathWorks, Inc., USA) and run on a computer with 16 GB RAM, Core i7, 11th generation processor.

The pH and TSS data were analyzed with Minitab 18.0 (Minitab Inc., USA) using a one-way analysis of variance (ANOVA) and the means were compared with a Tukey multiple comparison test with a significance level of 95 %.

## 3 Results

### 3.1 Evolution of physicochemical characteristics

**Fig. 2:**
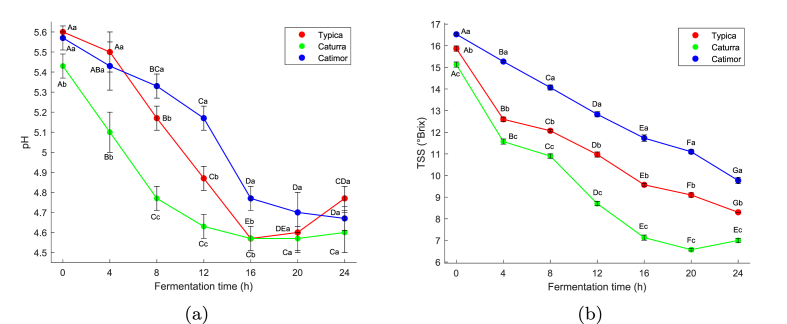
(a) Evolution of pH and (b) TSS during coffee fermentation Different capital letters indicate significant differences (*p* < 0.05) in the pH and TSS values of the same coffee variety (same color line) and different lowercase letters indicate significant differences (*p* < 0.05) between varieties. (For interpretation of the references to color in this figure legend, the reader is referred to the Web version of this article.)

Fig. 2(a) shows that the pH of the unfermented coffee beans was 5.60, 5.43, and 5.57 for the varieties Typica, Caturra, and Catimor, respectively. According to the National Institute of Agrarian Innovation in Peru, the pH of coffee for fermentation should be between 5.0 and 6.0 (Paredes Espinosa et al., 2022). At the same time, other authors stated that the optimal pH should be around 5.0 and 5.6 (Galarza and Figueroa, 2022; Córdoba-Castro and Guerrero-Fajardo, 2016). At the end of fermentation, the pH of the Typica, Caturra and Catimor coffee decreased to 4.77, 4.60, and 4.67, respectively. From the beginning until 16 h of fermentation, significant differences (*p* < 0.05) were evident in the pH values within each coffee variety but stabilized after 20 h.

The decrease in pH in the mass of coffee is attributed to the degradation of complex organic substances in mucilage to simpler sugars and consequent production of acids (Elhalis et al., 2023). After 16 h of fermentation, the decreasing and increasing trend of pH suggests that the increase in acid and alcohol production stopped fermentation (Sunoj et al., 2016). Similar results were found when evaluating Arabica coffee from the Catuaí Vermelho variety (pH from 5.7 to 4.5) in Brazil (de Jesus Cassimiro et al., 2022), Caturra (5.5–4.4) and Castillo (5.6–4.4) varieties in Colombia (Córdoba-Castro and Guerrero-Fajardo, 2016), and Sln 795 (5.6–4.2) variety in India (Shankar et al., 2022).

The evolution of pH between coffee varieties was significant (*p* < 0.05) in the first half of the process and subsequently the variation decreased until stability was reached. Similar trends were observed during the fermentation of Caturra and Castillo varieties (Córdoba-Castro and Guerrero-Fajardo, 2016), as well as Mundo Novo, Ouro Amarelo, and Catuaí Vermelho varieties (Ribeiro et al., 2018), and Tabi, Castillo General, and Colombia varieties (Holguín-Sterling et al., 2023). These differences reflect variations in the initial chemical composition of coffee cherries, the microbial activity, and the specific interactions of each variety with its fermentation environment.

Coffee varieties significantly influence the diversity of the microbiota and the formation of metabolites during fermentation, which is reflected in the disparity of pH values (Ribeiro et al., 2018). Holguín-Sterling et al. (2023) found notable differences in microbial diversity among coffee varieties during fermentation, with this diversity decreasing towards the end of the process. Furthermore, Peñuela-Martínez et al. (2023) confirmed these observations, showing significant differences in pH during the early stages of fermentation.

Since a final pH close to 4.6 was established to indicate the end of the fermentation process (Córdoba-Castro and Guerrero-Fajardo, 2016; Jackels and Jackels, 2005), an important point is that at 16 h, the pH of Typica and Caturra coffee ranged from 4.6, unlike Catimor coffee, which barely reached 4.67 after 24 h of fermentation. Pothakos et al. (2020) reported microbial diversity is restricted at pH levels lower than 4.5–4.0 during long fermentation, where the communities of lactic acid bacteria tolerant to the acidic environment prevail, affecting the flavor of the product. Under this premise, the fermentation time must be limited to 16 h.

Fig. 2(b) shows that the initial TSS of Typica, Caturra and Catimor coffee was 15.87, 15.13, and 16.53, respectively, similar to those reported by de Jesus Cassimiro et al. (de Jesus Cassimiro et al., 2022) around 16 °Brix, but lower than approximately 19.9 °Brix reported by Galarza and Figueroa (2022), Puerta and Echeverry (2015). So, Galarza and Figueroa (2022) indicated that coffee fruits should be harvested in a 12–24 °Brix range for adequate processing.

During fermentation, there was a reduction in TSS as a function of time, with significant differences (*p* < 0.05) between each variety, indicating the use of sugar by microorganisms. Until 16 h, a notable reduction in TSS was observed, similar to that described by Prakash et al. (2022) when evaluating Robusta coffee fermentation. According to the author, it could be attributed to the decomposition of complex carbohydrates into monosaccharides by the metabolic activity of yeast. As fermentation is prolonged, the population of yeast decreases due to the conversion of metabolites to alcohols and the consequent growth of lactic acid bacteria that continue to consume sugars, but at a slower rate (Prakash et al., 2022).

Significant differences (*p* < 0.05) were also observed in the evolution of TSS between coffee varieties, attributed to the composition and predominance of microorganisms during fermentation. Similar results were reported in the dynamics of certain sugars and acids (Holguín-Sterling et al., 2023; Peñuela-Martínez et al., 2023; Ribeiro et al., 2018).

Other factors influencing differences in characteristics, such as pH and compounds associated with TSS between coffee varieties during fermentation, include variations in the thickness of the mucilage layer (Velmourougane, 2013), as well as differences in the size and density of the beans (Peñuela-Martínez et al., 2023).

**Fig. 3:**
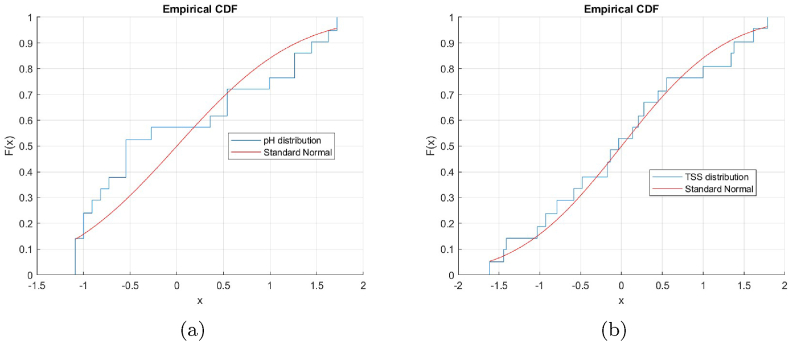
Normal distribution function vs distribution of (a) pH and (b) TSS.

From normality analysis (Kolmogorov-Smirnov test), *p* values of 1.7 × 10^−108^ and 3.9 × 10^−21^ were determined for pH and TSS values, respectively. This is consistent with what is shown in Fig. 3 for both parameters.

### 3.2 NIR spectral profiles

Table 1 shows the complete spectral profiles of the three coffee varieties and their relationship with pH and TSS. The shape of the spectral profile is similar to that described in Araújo et al. (2020), Chakravartula et al. (2022), Tugnolo et al. (2021) for ground and roasted coffee. Regarding the relevant peaks, there were slight differences in absorbance in the region of 1550–1800 nm that could be associated with variations in the content of chlorogenic acid, carbohydrates, amino acids, and caffeine (Munyendo et al., 2021), but the trends remained invariable.

**Table 1:**
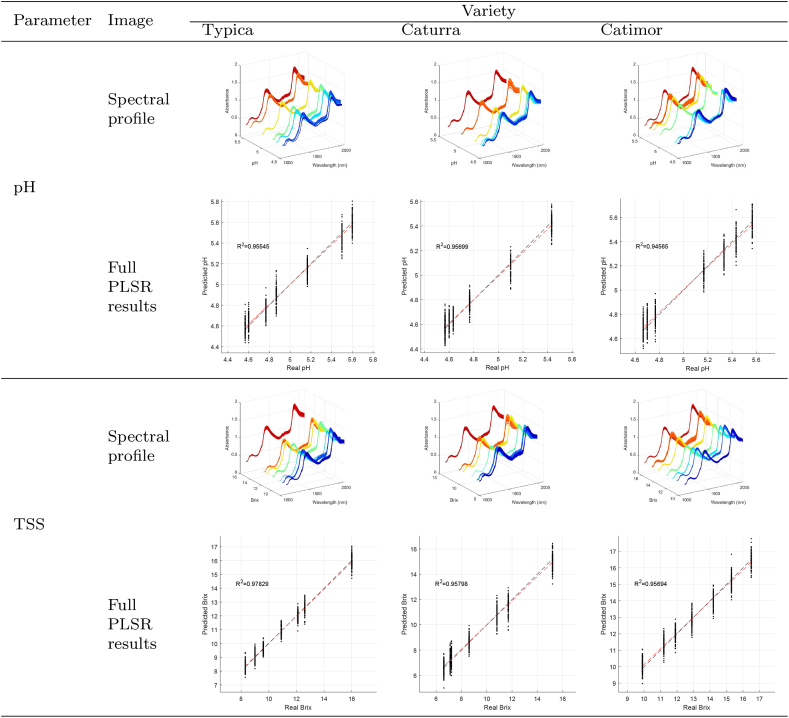
Spectral profiles and Full PLSR results per variety and physicochemical parameter.

The peak at 1205 nm is attributed to *C–H* band vibrations corresponding to fatty acids, amino acids, and lignin (Munyendo et al., 2021). Oktavianawati et al. (2020) revealed the presence of different amino acids in coffee beans, whose concentrations increased after fermentation. Shen et al. (2023) found 209 fatty acids in coffee beans subjected to different fermentation processes. Lignin is one of the main compounds in the endocarp or parchment of coffee beans. This peak is also associated with the second harmonic of *C–H* stretching corresponding to carbohydrates, lipids, and quinic acid (Munyendo et al., 2021). Sugars and lipids were determined to be characteristic markers of coffee fermentation (Agnoletti et al., 2022). Hu et al. (2020) showed that quinic acid and the coffee body score have a positive relationship. Shen et al. (2023) found 527 lipids in coffee beans, which retain the volatile flavor compounds and vitamins that contribute to texture and mouthfeel.

As expected, the predominant peaks at 1451 and 1927 nm are associated with the high water content in the coffee samples (Araújo et al., 2020; Tugnolo et al., 2021). They are also found in the region associated with the content of carbohydrates and chlorogenic acid (Munyendo et al., 2021), the latter responsible for the bitterness and astringency of coffee (Munyendo et al., 2021; Shen et al., 2023). The peaks at 1729 and 1927 nm are associated with the *C–H* harmonic bands of caffeine (Chakravartula et al., 2022; Sim et al., 2023); similarly, at 1839 nm, the *C–H* bonds could refer to cellulose, another predominant polysaccharide in the coffee endocarp (Sim et al., 2023).

### 3.3 Full PLSR models

Table 1 also displays the performance of the full PLSR models reflected in high *R*^2^ values (from 0.946 to 0.978). Regarding RMSE values in the pH prediction, low values between 0.064 and 0.082 were obtained, while in the TSS prediction, the dispersion of the data was greater with RMSE values of 0.364–0.600. At the same time, RPD, except for the TSS prediction model in Caturra coffee (RPD = 1.67), ranges from 2.25 to 15.55. That means that predictions are possible and with high values a good adjustment is possible (Araújo et al., 2020).

The pH prediction models showed RPD values of 12.21, 12.48, and 15.55 for Typica, Catimor, and Caturra varieties, reflecting a greater adjustment than those obtained in other studies where the models NIR and PLSR were combined. Among these studies, the study of Priambodo et al. (2022) for the determination of the pH of cocoa beans during fermentation, obtained *R*^2^ from 0.63 to 0.70 and RMSE 0.28–0.31 or Sunoj et al. (2016) that in similar conditions fits similarly (*R*^2^ = 0.58–0.75 and RMSE = 0.26–0.35), with RPD between 1.52 and 2.05. Similar results were obtained for another fermented product, as in the study of Wu et al. (2015) those who predicted pH during Chinese rice wine fermentation *R*^2^ = 0.90, RMSE = 0.15 or Giovenzana et al. (2014) for pH during craft beer fermentation *R*^2^ = 0.38 − 0.89 and RMSE = 0.10–0.29.

According to Kasemsumran et al. (2023), the low performance of some results could be due to the complexity of biological processes that could be visualized in several overlapped absorption bands. At the same time, the low adjustment of PLSR and TSS could be due to a nonlinear relationship, which has been faced by da Silva Melo et al. (da Silva Melo et al., 2022), Zeng et al. (2024) through nonlinear models.

### 3.4 Optimized PLSR models

Table 2 shows the wavebands selected by the proposed CAFS method and the *β* coefficients and VIP methods. For pH prediction high *R*^2^ values (see Table 3, Table 4) were obtained using VIP (> 0.921), *β* coefficients (> 0.749), and CAFS (> 0.825) approaches. Although RPD values varied in the ranges [9.15–11.56], [6.28–6.69], and [6.33 to 10.38], respectively.

**Table 2:**
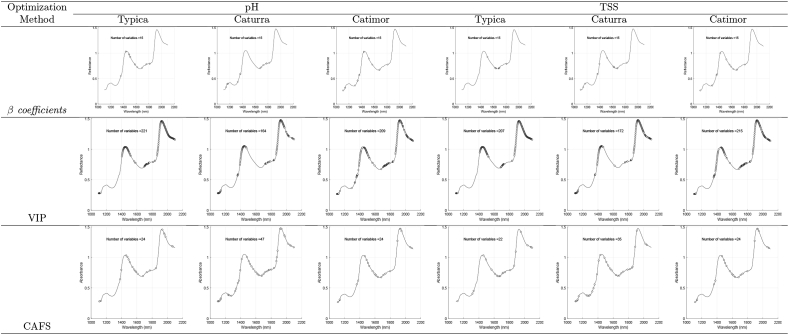
Selected features per each method.

**Table 3:**
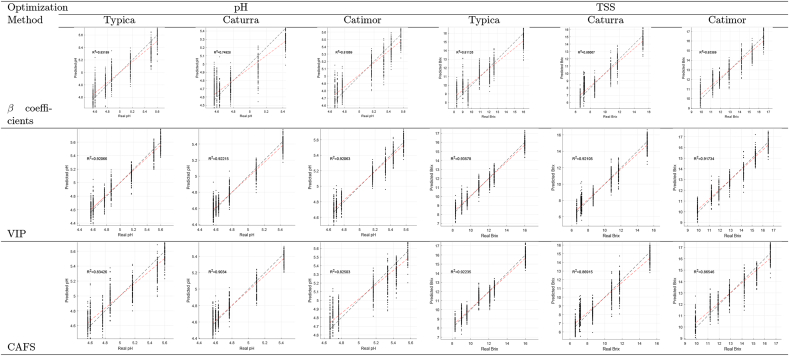
Optimized PLSR results per each method.

**Table 4:** Performance metrics of the full and optimized PLSR models.

PLSR			Typica			Caturra			Catimor		
			*R*^2^	RMSE	RPD	*R*^2^	RMSE	RPD	*R*^2^	RMSE	RPD
pH	Full	VIP	0.955	0.082	12.21	0.957	0.064	15.55	0.946	0.080	12.48
			0.921	0.109	9.15	0.922	0.087	11.56	0.921	0.097	10.33
	Opt	*β* coefficients	0.832	0.159	6.28	0.749	0.155	6.44	0.811	0.150	6.69
		CAFS	0.834	0.158	6.33	0.903	0.096	10.38	0.825	0.144	6.96
TSS	Full	VIP	0.978	0.364	2.75	0.958	0.600	1.67	0.957	0.445	2.25
			0.936	0.626	1.60	0.921	0.823	1.22	0.917	0.617	1.62
	Opt	*β* coefficients	0.811	1.073	0.93	0.861	1.093	0.92	0.854	0.820	1.22
		CAFS	0.922	0.688	1.45	0.869	1.059	0.94	0.865	0.787	1.27

The results, see Table 3, were better than in other studies using different VS approaches. In the study of Li et al. (2018) using GA and SPA to predict the pH of cherries with 24 and 28 variables, respectively, achieving *R*^2^ values of 0.819 and 0.815, RMSE, and RPD 2.351 and 2.326. Cheng et al. (2022) used the CARS, SPA, and UVE to predict the pH of pears with 52, 11, and 557 variables, respectively, achieving *R*^2^ values of 0.862–0.915 and an RMSE of 0.024–0.033. Lamptey et al. (2023) used the iPLS, Si-PLS, and Bi-PLS to predict the pH of mangoes with 52, 11, and 557 variables, respectively, achieving *R*^2^ values of up to 0.79–0.89, RMSE of 0.44–0.51 and RPD of 2.20–2.97.

Table 4 summarizes the statistical metrics; it is shown that the optimized models had a comparatively worse adjustment when TSS was predicted. The VIP, *β* coefficients, and the CAFS approach yielded *R*^2^ > 0.811, and RMSE from 0.626 to 1.093. Likewise, the instability of the models is reflected in values of excessive variation RPD of 1.22–1.62 for VIP, 0.92 to 1.22 for *β* coefficients, and 0.94 to 1.45 for CAFS. Saavedra et al. (2014) obtained similar results when predicting the TSS of cape gooseberry using iPLS, GA, rPLS and CovSel and obtained *R*^2^ of 0.48–0.58, RMSE of 0.61–0.61 and RPD of 1.61–1.77. Lamptey et al. (2023) used the iPLS, Si-PLS and Bi-PLS to predict the TSS of mangoes with 52, 11 and 557 variables, respectively, achieving *R*^2^ values of up to 0.60–0.67, RMSE of 1.85–1.94 and RPD of 1.58–1.74. Li et al. (2018) used the GA and SPA to predict TSS of cherries with 54 and 28 variables, respectively, achieving *R*^2^ values of 0.863 and 0.771, RMSE of 1.210 and 1.563 and RPD of 2.700 and 2.089. Superior results were obtained by Cheng et al. (2022) using CARS, SPA and SVU to predict the TSS of pears with 70, 18 and 505 variables, respectively, achieving *R*^2^ values of 0.876–0.943, RMSE of 0.142–0.248.

Regarding fermentation monitoring, Kasemsumran et al. (2022) used SCMWPLS to optimize the models and obtain *R*^2^ values of 0.996, RMSE of 0.166 and RPD of 27.82 in the prediction of TSS during the fermentation of pineapple wine. Despite the high predictive capacity, the model still contained a large number of variables (181); although it is applicable in a laboratory for experimental purposes, it is not very viable for practical purposes that involve the development of portable or online devices. Kasemsumran et al. (2023) used the same spectrometer to monitor TSS in pineapple vinegar broth during fermentation. The model was optimized with only 21 variables using stability CARS (SCARS), obtaining an *R*^2^ of 0.903 and an RMSE of 0.875.

Regarding the effect of each VS method on the metrics of the PLSR models, VIP obtained the best results, but selected a large number of variables (from 164 to 221) compared to CAFS (from 22 to 47) and *β* coefficients (15). The selection of variables with VIP was concentrated at some points (see Table 2), which could have been the product of the codependence of the variables. Although the results were comparable to those obtained with the full spectrum, the model is not viable for practical applications. Similarly, in Liao et al. (2012), the VIP-PLSR method to predict pH in fresh beef selected 20.13% of the variables but showed similar results to when all variables were used. Similar trends were reported when using VIP to predict the same parameters (Afonso et al., 2022; Cevoli et al., 2024). The VIP could have selected many variables because it focuses on the weight of the variable in the projection but ignores its stability. Some variables whose stability was the same as the noise played an important role within the latent projection but provided a performance deficiency in the PLSR model (Liao et al., 2012).

Regarding *β* coefficients, Chong and Jun determined that, in terms of quantitative selection of relevant predictors, the PLSR-*β* coefficients method is superior to the PLSR-VIP method, which was corroborated in this study. However, the performance of the PLSR-*β* coefficients models for the prediction of TSS was low (RPD of 0.92–1.22) compared to the estimation of pH (RPD of 6.28–6.69). According to Cattaldo et al. (2024), the *β* coefficients work well even for small samples, but when the true complexity of the model is linear, they are less suitable for modeling nonlinear problems, as discussed above, regarding the relationship between the spectra and TSS.

The *β* coefficients and CAFS approaches were comparable in terms of the number of variables selected, but, as expected, the latter showed better performance. With the *β* coefficients approach, variables were selected in certain bands, limiting themselves to local minima, while CAFS tried to select relevant variables throughout the spectrum. In this way, the problem of modeling TSS was also improved. TSS involve several compounds such as vitamins, sugars, and amino acids, so it is difficult to assign importance to a single region of the spectrum, as reported by Agulheiro-Santos et al. (2022) when predicting TSS in strawberries. In some cases, the search for variables throughout the spectral region is not desirable because it can lead to the potential problem of placing unrelated variables, as reported in the prediction of TSS in tomatoes (Li et al., 2020). This was addressed thanks to the maximum coverage and minimum cardinality of CAFS. Similar results were obtained when using CAFS to discriminate Amazonian cacao-clone nibs (Castro et al., 2022).

The results highlighted the potential of CAFS to select effective wavelengths in the NIR spectrum and develop robust PLSR models to predict the evolution of pH and TSS during coffee fermentation. Coffee producers could replicate the findings of this study to avoid excessive or incomplete fermentations, which would represent a great transition step from rural to technology with the aim of industrial scale and adoption.

## 4 Conclusions

This study proposed using CAFS, a new VS approach to regression problems based on coverage matrices. CAFS was tested to predict the evolution of the physicochemical parameters during the fermentation of three varieties of coffee using NIR spectra and PLSR models. Unlike common algorithms such as VIP and *β* coefficients, the models with wavelengths selected by CAFS showed the best performance in pH prediction (*R*^2^ = 0.825–0.903, RMSE = 0.096–0.158, RPD = 6.33–10.38) and TSS (*R*^2^ = 0.865–0.922, RMSE = 0.688–1.059, RPD = 0.94–1.45). CAFS selected limited but important variables (22–47) throughout the spectrum due to their maximum coverage and minimum cardinality. This is convenient in practice because it allows the development of low-cost miniaturized systems for real-time monitoring of the process, facilitating the production of quality coffee for small coffee growers. The performance of the models can be improved by increasing the number of samples, testing nonlinear models, and using different features or CA families. Furthermore, research can potentially be extended to other quality parameters and applied to other food processes and products.

## Declaration of competing interest

The authors declare the following financial interests/personal relationships which may be considered as potential competing interests:

Wilson Castro reports financial support was provided by Programa Nacional de Investigación Científica y Estudios Avanzados (PROCIENCIA, Peru), Project "Sistema prototipo de determinación de calidad de taza para café: estudio de técnicas deep learning", CONTRACT No. PE501080928-2022-PROCIENCIA. If there are other authors, they declare that they have no known competing financial interests or personal relationships that could have appeared to influence the work reported in this paper.

## Data Availability

Data will be made available on request.
